# Cylindrical Scan Context: A Multi-Channel Descriptor for Vertical-Structure-Aware LiDAR Localization

**DOI:** 10.3390/s25237223

**Published:** 2025-11-26

**Authors:** Chulhee Bae, Gun Rae Cho, Jongho Bae, Sungho Park, Mangi Lee, Shin Kim, Jung Hyeun Park

**Affiliations:** Korea Institute of Robotics & Technology Convergence, Pohang 37666, Republic of Korea; sandman@kiro.re.kr (G.R.C.); jongho.bae@kiro.re.kr (J.B.); psh84@kiro.re.kr (S.P.); mangi11@kiro.re.kr (M.L.); mki963@kiro.re.kr (S.K.); junghyeun0280@kiro.re.kr (J.H.P.)

**Keywords:** LiDAR, place recognition, loop closure, scan context, cylindrical descriptor, SLAM

## Abstract

This study introduces Cylindrical Scan Context (CSC), a novel LiDAR descriptor designed to improve robustness and efficiency in GPS-denied or degraded outdoor environments. Unlike the conventional Scan Context (SC), which relies on azimuth–range projection, CSC employs an azimuth–height representation that preserves vertical structural information and incorporates multiple physical channels—range, point density, and reflectance intensity—to capture both geometric and radiometric characteristics of the environment. This multi-channel cylindrical formulation enhances descriptor distinctiveness and robustness against viewpoint, elevation, and trajectory variations. To validate the effectiveness of CSC, real-world experiments were conducted using both self-collected coastal–forest datasets and the public MulRan–KAIST dataset. Mapping was performed using LIO-SAM with LiDAR, IMU, and GPS measurements, after which LiDAR-only localization was evaluated independently. A total of approximately 700 query scenes (1 m ground-truth threshold) were used in the self-collected experiments, and about 1200 scenes (3 m threshold) were evaluated in the MulRan–KAIST experiments. Comparative analyses between SC and CSC were performed using Precision–Recall (PR) curves, Detection Recall (DR) curves, Root Mean Square Error (RMSE), and Top-*K* retrieval accuracy. The results show that CSC consistently yields lower RMSE—particularly in the vertical and lateral directions—and demonstrates faster recall growth and higher stability in global retrieval. Across datasets, CSC maintains superior DR performance in high-confidence regions and achieves up to 45% reduction in distance RMSE in large-scale campus environments. These findings confirm that the cylindrical multi-channel formulation of CSC significantly improves geometric consistency and localization reliability, offering a practical and robust LiDAR-only localization framework for challenging unstructured outdoor environments.

## 1. Introduction

Accurate and reliable localization is a critical component in autonomous driving, robotic navigation, and maritime or port mobility systems. Although satellite-based navigation systems such as the Global Positioning System (GPS) provide dependable global positioning in open-sky environments, their performance can degrade severely in unstructured or cluttered outdoor areas—such as coastal regions, mountainous terrain, or dense forests—due to multipath distortion, signal occlusion, or intentional interference such as jamming and spoofing. These limitations have motivated increased interest in LiDAR-based localization, which leverages the rich geometric structure of the environment and remains functional even under GPS-denied or degraded conditions.

LiDAR sensors provide dense three-dimensional measurements that enable robust pose estimation in challenging outdoor environments [1,2]. Traditional registration-based methods, including Iterative Closest Point (ICP) and Normal Distribution Transform (NDT), estimate relative motion by aligning consecutive point clouds. While such approaches can yield high accuracy, their computational demand and sensitivity to initialization make them unsuitable for large-scale, real-time applications. To address these challenges, global descriptor–based localization methods have been proposed, transforming raw point clouds into compact and distinctive representations for fast place recognition. One notable descriptor is Scan Context (SC) [3], which discretizes a LiDAR scan into a ring–sector grid on an azimuth–range plane for efficient similarity computation.

Despite its advantages, SC encodes the vertical (*z*-axis) structure of each grid cell using only a single maximum height value ($z_{\max}$), inevitably discarding multi-layered height information. Therefore, in environments characterized by complex vertical geometry—such as forested areas with overlapping canopy layers, coastal cliffs with large elevation gradients, or hilly terrain—SC may fail to capture subtle geometric distinctions, reducing its discriminability and robustness to viewpoint changes.

To overcome these limitations, this study introduces the Cylindrical Scan Context (CSC), a novel LiDAR descriptor that represents each scan in an azimuth–height domain rather than the traditional azimuth–range plane. CSC discretizes the space along the vertical axis, preserving multi-level height variations and capturing richer geometric structures. In addition, CSC integrates multiple physical channels—including maximum range, point density, and reflectance intensity—to jointly encode geometric and radiometric information [4]. This multi-channel cylindrical formulation enhances distinctiveness, improves robustness against viewpoint and trajectory variations, and aligns with recent trends toward multi-modal and semantically aware LiDAR localization [5,6]. Beyond descriptor design, this work also addresses a key operational challenge in LiDAR-only localization: how to convert global descriptor retrieval into an accurate final pose estimate. CSC provides coarse global place candidates that are robust even under large viewpoint differences; however, descriptors alone cannot yield a full 6-DoF pose. Therefore, we adopt a two-stage localization framework in which (1) CSC performs global retrieval to propose high-confidence pose candidates, and (2) ICP refines these candidates locally by aligning the query scan to a submap. In this pipeline, CSC handles global search and viewpoint invariance, while ICP provides fine-grained geometric alignment, enabling the system to produce accurate and consistent localization results even in complex environments.

The main contributions of this work are summarized as follows:

- We construct a real-world dataset in a mixed coastal–forest environment and empirically validate the responsiveness and reliability of LiDAR-only localization using a pre-built SLAM map.
- We develop a two-stage localization framework consisting of (i) CSC-based global retrieval, which generates coarse pose hypotheses, and (ii) ICP-based local pose refinement, which accurately aligns the query scan with the local map.
- We present comprehensive comparisons between SC and CSC under identical conditions and quantitatively evaluate their performance using Precision–Recall (PR), Detection Recall (DR), Root Mean Square Error (RMSE), and Top-*K* retrieval accuracy metrics.

It is important to clarify that the focus of this work is not on improving SLAM-based map construction, but rather on evaluating operational performance of LiDAR-only localization when revisiting a pre-built map under real-world conditions. To this end, mapping and localization were performed in separate sessions, demonstrating the robustness of CSC under temporal variations, viewpoint changes, and heterogeneous traversal trajectories.

The remainder of this paper is organized as follows: Section 2 reviews related works on LiDAR-based localization; Section 3 describes the proposed CSC methodology; Section 4 presents experimental results and quantitative analyses; and Section 5 concludes the paper with discussions on scalability and future research directions.

## 2. Related Works

LiDAR-based localization methods can generally be divided into two categories: registration-based approaches and descriptor-based global localization [7]. This section reviews the characteristics and recent trends of both approaches and explains the motivation behind the proposed Cylindrical Scan Context (CSC).

### 2.1. Registration-Based Approaches

Registration-based methods estimate the relative pose between two LiDAR scans by directly aligning their geometric correspondences. The classic Iterative Closest Point (ICP) algorithm [8] remains a cornerstone technique that iteratively minimizes point-to-point distance errors between two point clouds. Although ICP provides high accuracy, it is highly sensitive to the initial pose estimation and may diverge in sparse or noisy observations. To enhance its robustness, probabilistic and feature-based registration variants have been proposed, such as the Normal Distributions Transform (NDT) [9,10,11], which models local surfaces as Gaussian distributions for stable convergence, and local feature-based matching that extracts keypoints to improve computational efficiency. Nevertheless, these methods remain computationally intensive and struggle to scale in large or dynamic environments, making them less suitable for real-time applications on embedded or resource-limited platforms.

### 2.2. Descriptor-Based Global Localization

In contrast, descriptor-based global localization aims to transform point clouds into compact, rotation-invariant representations that allow fast database retrieval and loop closure detection. These approaches have attracted growing attention because they offer an advantageous balance between accuracy, efficiency, and scalability in large-scale outdoor mapping.

A representative method is the Scan Context (SC) [3], which projects a LiDAR scan into a ring–sector grid in polar coordinates and measures similarity through efficient matrix comparison. Following its introduction, numerous extensions have sought to enhance robustness, distinctiveness, and adaptability to different environments. For instance, M2DP [12] projects a point cloud from multiple viewpoints into 2D matrices and applies Singular Value Decomposition (SVD) to achieve rotation invariance. Intensity Scan Context (ISC) [13] incorporates reflectance intensity information to improve discrimination in geometrically repetitive areas, while Scan Context++ [14] strengthens rotational and revisiting robustness in urban driving scenarios.

More recently, several learning- and semantics-based approaches have expanded the capability of LiDAR localization. LISA (LiDAR Localization with Semantic Awareness) [5] integrates semantic segmentation through attention-guided feature fusion, improving recognition under illumination or viewpoint changes. DiffLoc [6] introduces diffusion probabilistic models to learn continuous pose distributions, achieving better generalization to unseen outdoor environments. Meanwhile, Zhao et al. [15] propose a Monte Carlo distortion simulation framework for multi-sensor LiDAR data augmentation, significantly improving robustness against motion distortion and sensor noise. Other learning-based works, including OverlapNet [16], LiDAR Iris [17], and MixedSCNet [18], further explore deep representations and multi-modal fusion for long-term and cross-domain localization.

Together, these studies mark a clear transition toward semantically enriched, data-driven, and multi-channel LiDAR localization frameworks. However, most of them still rely on azimuth–range projections, which inherently simplify the vertical (*z*-axis) dimension, leading to reduced discriminability in environments with complex elevation variations.

### 2.3. Limitations of Existing Methods

Although descriptor-based methods have achieved remarkable progress in improving localization efficiency and robustness, they generally suffer from the loss of fine-grained vertical information. In SC, for example, only the maximum height value is stored within each ring–sector bin, limiting its representation of complex vertical structures in natural scenes. Moreover, because the polar coordinate system’s bin area increases proportionally with radial distance, distant regions contain more points and lose local detail due to averaging effects. This leads to degraded discriminability in far-range or high-elevation regions such as forests, slopes, and cliffs. Although structured urban environments contain prominent vertical landmarks that may help mitigate some of SC’s limitations, unstructured environments with irregular elevation and vegetation still lead to reduced recall. These limitations highlight the necessity for a representation that uniformly captures 3D structural variations across both height and range, without introducing geometric bias.

### 2.4. Motivation for CSC

Motivated by these challenges, this study proposes the Cylindrical Scan Context (CSC). CSC divides the surrounding space into an azimuth–height grid, maintaining uniform height intervals and constant bin areas independent of radial distance. This design enables CSC to preserve vertical information that SC discards while achieving balanced spatial resolution across both near and far regions. As a result, CSC effectively captures natural 3D features such as terrain elevation, slopes, cliffs, and vegetation heights, enhancing global distinctiveness and localization stability in unstructured environments. In the proposed framework, CSC performs efficient global candidate retrieval, followed by ICP-based refinement for precise pose estimation. By combining CSC’s descriptive richness with ICP’s geometric precision, the proposed two-stage pipeline achieves robust LiDAR-only localization even in GPS-denied or degraded conditions.

## 3. Methodology

In this section, the algorithmic architecture and operational procedure of the proposed Cylindrical Scan Context (CSC)-based LiDAR-only localization framework are presented. First, the conceptual design and descriptor generation process of CSC are defined, followed by a step-by-step description of the localization pipeline. Finally, computational efficiency and implementation considerations are briefly discussed.

### 3.1. Concept and Descriptor Generation

The conventional Scan Context (SC) projects a point cloud onto a polar coordinate system, representing it as a two-dimensional matrix on the azimuth–range plane, where each cell records the maximum height value ($z_{\max}$) within its region. This approach achieves high computational efficiency and partial invariance to yaw rotation, but it fails to sufficiently capture structural information along the vertical direction or physical properties such as reflection intensity.

To overcome these limitations, this study extends the concept of SC and proposes the Cylindrical Scan Context (CSC), which employs an azimuth–height representation instead of the traditional azimuth–range projection. CSC converts the point cloud into cylindrical coordinates (r,θ,z) and records multiple statistical channels—including maximum range, point density, and intensity—on a two-dimensional grid with azimuth θ and height *z* axes. Through this design, CSC encodes both the geometric and radiometric characteristics of the environment simultaneously. Moreover, by discretizing along the height (*z*) axis, each bin in CSC maintains a constant spatial area independent of radial distance (*r*), thus providing a uniform spatial resolution across all ranges. This alleviates the over-expansion of outer ring bins in SC, which often causes the loss of fine structural details in distant regions.

Each channel captures distinct physical attributes: the range channel (*r*) quantifies spatial distribution relative to the sensor origin; the density channel expresses the concentration of points within a given region, allowing for effective discrimination of vertical structures or obstacles; and the intensity channel reflects surface reflectivity, enabling distinction between objects with similar geometry but different material or roughness properties. By integrating these channels, CSC provides a richer and more discriminative structural representation compared to the single-height encoding of SC.

Since CSC constructs its descriptor on the azimuth–height plane, it inherently preserves partial rotational robustness to yaw variation. In other words, even when the sensor’s viewing direction changes, the cylindrical projection ensures that similar descriptors are generated for the same environment. This design allows CSC to retain vertical structural information (e.g., trees, slopes, cliffs) while maintaining intensity and density distributions, resulting in more stable place recognition performance in unstructured natural environments.

**Figure 1:**
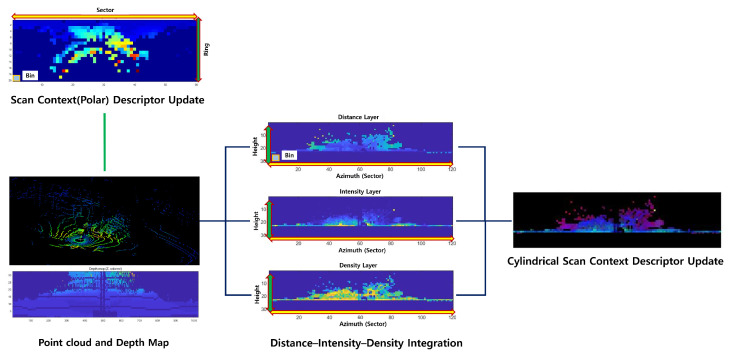
Comparison between SC and CSC. CSC preserves vertical structural information by encoding multiple channels—maximum range, point density, and intensity on an azimuth–height grid.

Figure 1. conceptually compares the structural difference between SC and CSC. While SC consists of a single-channel representation, CSC incorporates multiple channels on the azimuth–height plane, providing a richer and more expressive environmental description.

### 3.2. Localization Algorithm Architecture

**Figure 2:**
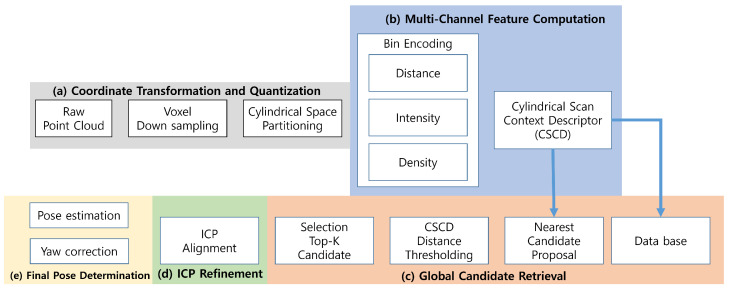
CSC-based localization pipeline. After coordinate transformation and CSC generation, the system determines the final pose through global retrieval and ICP refinement.

The proposed CSC-based LiDAR-only localization algorithm consists of five main stages, as illustrated in Figure 2. (a) Coordinate transformation and quantization convert the input point cloud into cylindrical coordinates. (b) CSC generation constructs a multi-channel descriptor. (c) Global retrieval searches for candidate locations in the pre-built database. (d) Candidate ICP refinement performs precise pose alignment. Finally, (e) the best-matching pose is selected as the LiDAR-only global localization result.

(a) Coordinate Transformation and Quantization

Each point p=(x,y,z) is transformed into the cylindrical coordinate system:

(1) $$r=\sqrt{x^{2}+y^{2}},\;\theta =\mathrm{atan}2\left(y,x\right),\;z=z$$

The azimuth θ∈[0,2π) and height $z\in [z_{\min},z_{\max}]$ are uniformly quantized into $N_{\theta }$ and $N_{z}$ bins, respectively. Each point is mapped to its corresponding indices as follows:

(2) $$b_{\theta }=\left\lfloor \frac{\theta }{2\pi }N_{\theta }\right\rfloor ,\;b_{z}=\left\lfloor \frac{z-z_{\min}}{z_{\max}-z_{\min}}N_{z}\right\rfloor$$

(b) Multi-Channel Feature Computation

For each bin $\left(b_{\theta },b_{z}\right)$, the proposed CSC descriptor extracts three statistical features from the corresponding set of points $P_{b_{\theta },b_{z}}$. Specifically, the feature vector is defined as

(3) $$a_{b_{\theta },b_{z}}={\left[r_{\max},\;\mathrm{dens},\;\bar{I}\right]}^{⊤}\in R^{3},$$

where $r_{\max}$ is the maximum range within the bin, dens denotes the point density, and $\bar{I}$ represents the mean intensity. These three channels provide a compact yet effective representation of geometric and radiometric characteristics: the maximum range encodes the structural extent of objects, density reflects the occupancy level of local regions, and intensity provides additional radiometric contrast.

The complete CSC descriptor is stored as a 3D tensor:

(4) $$D\in R^{N_{z}\times N_{\theta }\times 3}.$$

For efficient global retrieval, the channel dimension and height bins are flattened into a matrix representation

$$\hat{D}\in R^{N_{h}\times N_{\theta }},\;N_{h}=3\cdot N_{z},$$

which allows cosine-distance comparison with circular column shifts.

(c) Global Candidate Retrieval

The query point cloud $P_{q}$ is encoded into a descriptor ${\hat{D}}_{q}$, which is compared against all database entries $\mathrm{DB}={\left\{\left({\hat{D}}_{k},T_{k}\right)\right\}}_{k=1}^{N}$. The similarity is evaluated using cosine distance with circular column-wise shifts:

(5) $$d\left({\hat{D}}_{q},{\hat{D}}_{k}\right)=\min_{\Delta \theta \in \{0,\ldots ,N_{\theta }-1\}}\left(1-\frac{\langle {\hat{D}}_{q},.{\mathrm{shift}}_{\Delta \theta }\left({\hat{D}}_{k}\right)\rangle }{∥{\hat{D}}_{q}∥\cdot ∥{\hat{D}}_{k}∥}\right)$$

where ${\mathrm{shift}}_{\Delta \theta }\left(\cdot \right)$ denotes a circular shift along the azimuth direction. The optimal shift $\Delta \theta^{*}$ corresponding to the minimum distance *d* is converted to a yaw correction term:

(6) $$\Delta \psi =-\Delta \theta^{*}\cdot \frac{2\pi }{N_{\theta }}$$

Based on the computed distances, the Top-*K* candidate indices $C=\{k_{1},\ldots ,k_{K}\}$ are selected. For each candidate, the initial pose is computed as

(7) $$T_{k}^{\left(0\right)}=T_{k}\cdot R_{z}\left(\Delta \psi_{k}\right),\;k\in C$$

(d) Candidate ICP Refinement

For the selected Top-*K* candidates, a local map $M_{k}$ is built around each candidate position, typically within a radius of $r_{\mathrm{local}}=20$–30 m. Starting from the initial pose $T_{k}^{\left(0\right)}$, point-to-point ICP registration is performed:

(8) $$T_{k}^{★}=\arg\min_{T\in SE(3)}\sum_{\left(u,v\right)}{∥Tp_{u}-q_{v}∥}^{2}$$

The alignment iterates until convergence, defined by $∥\Delta T∥<\epsilon_{\mathrm{icp}}$ or a maximum iteration limit $N_{\max}$. Each candidate’s alignment quality is measured by a fitness score $J_{k}$, and the candidate with the smallest $J_{k}$ proceeds to the final selection stage.

(e) Final Pose Determination

Among all candidates, the result with the minimum fitness score below a threshold $\tau_{\mathrm{icp}}$ is selected as the final pose:

(9) $$\left(k^{*},T^{*}\right)=\arg\min_{k}J_{k},\;s.t.\;J_{k}<\tau_{\mathrm{icp}}$$

The final transformation $T^{*}$ represents the LiDAR-only global localization output, demonstrating stable and repeatable perform

## 3.3. Parameter Settings

To ensure reproducibility and enable fair comparison between SC- and CSC-based localization, all experimental parameters were consistently applied across both the self-collected and public datasets.

For the cylindrical projection, the 360° azimuth range was uniformly divided into $N_{\theta }=60$ bins, corresponding to an angular resolution of 6° per bin. The height axis was discretized over the range z∈[−10m,+20m], producing a total vertical span of 30 m. This configuration provides consistent spatial resolution regardless of radial distance, ensuring that distant structures receive the same vertical representation as nearby regions.

Each cell in the CSC descriptor stores a three-dimensional feature vector

$$a_{b_{\theta },b_{z}}={\left[r_{\max},\;\mathrm{dens},\;\bar{I}\right]}^{⊤},$$

as defined in Equation (3). A channel-wise weight vector

$$w=[w_{r_{\max}},\;w_{\mathrm{dens}},\;w_{I}]$$

was applied during descriptor flattening to balance the contribution of geometric ($r_{\max}$, dens) and radiometric ($\bar{I}$) cues. These weights were empirically tuned and fixed across all experiments to ensure consistent evaluation.

These weights were empirically tuned to enhance geometric cues (e.g., density, range) while preventing the intensity channel from dominating descriptor similarity. The same weight configuration was used for all datasets to ensure consistent evaluation.

For global retrieval, cosine distance with circular column shifts (Equation (5)) was used, and the system selected up to Top-K=30 candidates from the descriptor database. This large retrieval pool provides robustness against viewpoint variation, environmental noise, and partial occlusions.

Among the retrieved candidates, the top three were used for ICP-based pose refinement. For each candidate, a local submap with a radius of 20–30 m was extracted, and point-to-point ICP was executed until the convergence threshold or iteration limit was reached. The candidate producing the minimum ICP fitness score was chosen as the final localization result (Equation (9)).

All modules were implemented in C++ and executed on the target computing platform. With the complete pipeline—including cylindrical projection, descriptor generation, global retrieval, and ICP refinement—the system operated at approximately 2 Hz, providing sufficient responsiveness for low-speed autonomous navigation and static localization tasks.

## 4. Experimental

To comprehensively validate the proposed CSC-based LiDAR-only localization framework, experiments were conducted using two types of datasets: (1) a self-collected outdoor dataset acquired in a complex coastal–forest environment, and (2) the publicly available MulRan-KIAST [19] dataset containing repeated traversals of the same campus area. Together, these datasets allow evaluation under diverse conditions, including unstructured natural terrain, urban-like campus structures, temporal variations, and trajectory-dependent viewpoint changes. The complete workflow of the proposed method is summarized in Algorithm 1, which outlines the full CSC-based LiDAR-only global localization pipeline.

### 4.1. Self-Collected Dataset (Pohang, South Korea)

**Figure 3:**
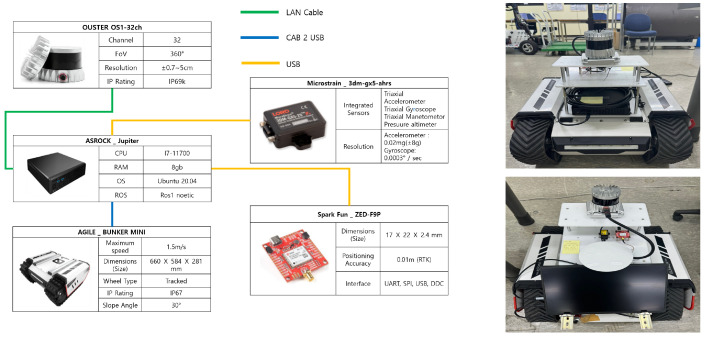
Configuration of the driving platform for data collection.

**Figure 4:**
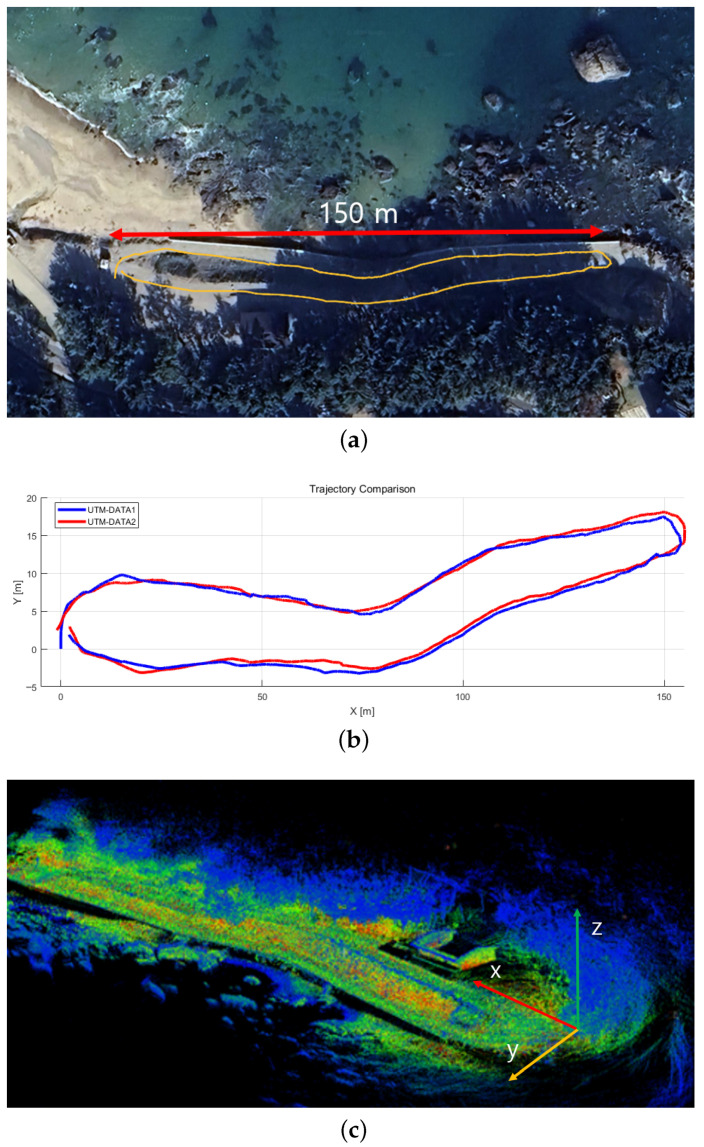
(**a**) Experimental environment (satellite imagery), (**b**) comparison of UTM trajectories, and (**c**) visualization of the mapping results. Since all maps share similar spatial structures, only representative visualizations are presented for clarity.

The first dataset was collected in a coastal–forest mixed terrain located in Pohang, South Korea. This region features elevation variations, curved paths, sloped road segments, and dense vegetation, making it a challenging environment for LiDAR-only localization. A sensing platform equipped with an Ouster OS1-32 LiDAR, a MicroStrain 3DM-GX5 IMU, and a SparkFun RTK-GPS module was driven along an outdoor route of approximately hl1 km. The detailed sensor platform configuration is shown in Figure 3, and the driving environment and UTM-referenced trajectories are illustrated in Figure 4a.

During the mapping stage, LiDAR, IMU, and GPS data were temporally synchronized and fused within the LIO-SAM framework to generate globally consistent maps. Both SC-based and CSC-based SLAM pipelines were executed independently, producing two sets of maps for each driving session. Descriptors extracted from individual LiDAR frames were stored together with their estimated poses to build the retrieval database.
**Algorithm 1** CSC-Based LiDAR-Only Global Localization.**Require:** 
Pre-built global map M, descriptor database $\mathrm{DB}={\left\{\left({\hat{D}}_{k},T_{k}\right)\right\}}_{k=1}^{N}$**Ensure:** 
Global LiDAR-only pose estimate $T^{*}$  1:Set descriptor parameters: $N_{\theta }=60$, z∈[−10,20]m  2:Set retrieval/ICP parameters: Top-K=30, M=3, $r_{\mathrm{local}}=20$–30m, threshold $\tau_{\mathrm{icp}}$  3:**for** each incoming query scan $P_{q}$ **do**  4:                          ▹ CSC descriptor generation  5:    Convert all points p=(x,y,z) in $P_{q}$ to cylindrical coordinates (r,θ,z)  6:    Quantize (θ,z) into bins $\left(b_{\theta },b_{z}\right)$ using $N_{\theta }$ and $\left[z_{\min},z_{\max}\right]$  7:    **for** each bin $\left(b_{\theta },b_{z}\right)$ **do**  8:        Collect point set $P_{b_{\theta },b_{z}}$  9:        Compute maximum range $r_{\max}$ in the bin10:        Compute point density dens and mean intensity $\bar{I}$11:        Form feature vector $a_{b_{\theta },b_{z}}={\left[r_{\max},\;\mathrm{dens},\;\bar{I}\right]}^{⊤}$12:    **end for**13:    Assemble CSC tensor $D_{q}$ and flatten to matrix form ${\hat{D}}_{q}$14:    Apply channel-wise weighting (if enabled)15:                   ▹ Global retrieval (Top-*K* candidates)16:    **for** each database entry $\left({\hat{D}}_{k},T_{k}\right)$ in DB **do**17:        Compute cosine distance with circular shifts $d_{k}=d\left({\hat{D}}_{q},{\hat{D}}_{k}\right)$18:        Store best shift $\Delta \theta_{k}^{*}$ associated with $d_{k}$19:    **end for**20:    Select Top-*K* candidates $C=\{k_{1},\ldots ,k_{K}\}$ sorted by ascending $d_{k}$21:                ▹ Initial pose hypotheses from yaw correction22:    **for** each k∈C **do**23:        Compute yaw correction $\Delta \psi_{k}=-\Delta \theta_{k}^{*}\cdot \frac{2\pi }{N_{\theta }}$24:        Set initial pose $T_{k}^{\left(0\right)}=T_{k}\cdot R_{z}\left(\Delta \psi_{k}\right)$25:    **end for**26:                   ▹ ICP refinement on Top-*M* candidates27:    $J_{\min}\leftarrow \infty$, $T^{*}\leftarrow I$28:    Select Top-*M* indices from C with the smallest $d_{k}$29:    **for** each selected candidate index *k* **do**30:        Extract local submap $M_{k}$ from M within radius $r_{\mathrm{local}}$ around $T_{k}^{\left(0\right)}$31:        Run ICP between $P_{q}$ and $M_{k}$ initialized at $T_{k}^{\left(0\right)}$32:        Obtain refined pose $T_{k}^{★}$ and fitness score $J_{k}$33:        **if** $J_{k}<J_{\min}$ **then**34:           $J_{\min}\leftarrow J_{k}$, $T^{*}\leftarrow T_{k}^{★}$35:        **end if**36:    **end for**37:                          ▹ Final pose validation38:    **if** $J_{\min}<\tau_{\mathrm{icp}}$ **then**39:        Accept $T^{*}$ as the final LiDAR-only global pose40:    **else**41:        Mark localization as invalid (e.g., keep previous pose or odometry-only estimate)42:    **end if**43:**end for**

To evaluate pure LiDAR-only localization, GPS measurements were intentionally removed during the localization stage. This design simulates realistic autonomous operation in GPS-denied outdoor environments, in which the mapping and localization stages occur at different times.

Two separate LiDAR–IMU–GPS sequences, denoted as data1 and data2, were collected by driving the same route on different occasions. Although collected in the same environment, these sequences exhibit natural differences in vegetation, lighting, and viewpoint. For each sequence, SC- and CSC-based SLAM were performed independently, resulting in four maps:

- SC-based maps: map1 (from data1), map2 (from data2);
- CSC-based maps: map1 (from data1), map2 (from data2).

Figure 4c shows a representative map. These maps were used to analyze the localization accuracy, robustness to temporal changes, and map reusability when the robot revisits the same environment under slightly different conditions.

### 4.2. Experiments Using Public Dataset (MulRan-KIAST)

To examine the generalizability and scalability of the proposed method, additional experiments were conducted using the publicly available MulRan-KIAST dataset. This dataset contains LiDAR–IMU recordings collected on the KAIST campus, where the same spatial region was traversed three times along different driving trajectories. Compared to the self-collected natural-terrain dataset, the KAIST environment includes a mix of structured buildings, pedestrian walkways, and vegetation, offering complementary evaluation conditions.

For the experiments:

- The second traversal was used as the reference mapping session, where SC-based and CSC-based SLAM were applied to build two maps.
- The first and third traversals were used exclusively for LiDAR-only localization testing against the pre-built maps.

**Figure 5:**
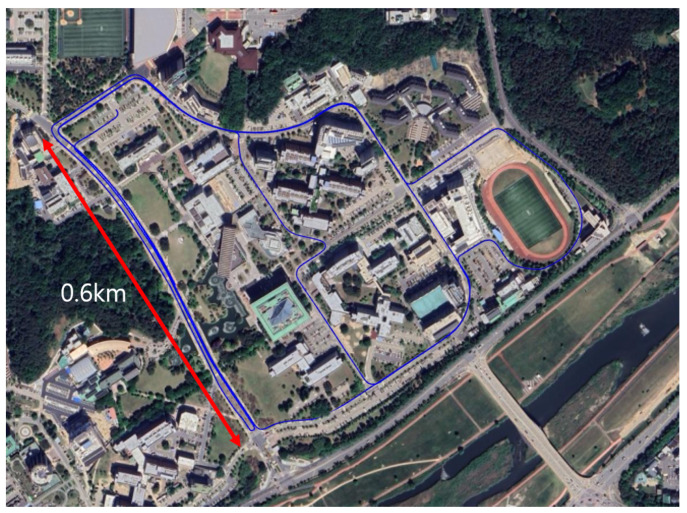
Visualization of the KAIST1 driving trajectory in the MulRan dataset overlaid on satellite imagery.

Because the three sequences cover the same areas while following different motion patterns, the dataset enables systematic analysis of viewpoint variation, loop-based localization stability, and map reusability. Figure 5 is visualization of the KAIST1 driving trajectory in the MulRan dataset overlaid on satellite imagery. By combining results from both the self-collected dataset and the MulRan-KIAST dataset, the proposed CSC framework is evaluated across diverse environments, natural and temporal variations, and trajectory-dependent perception differences—providing a comprehensive validation of its robustness and practicality.

### 4.3. Evaluation Metrics

To quantitatively evaluate the performance of the proposed Cylindrical Scan Context (CSC)-based localization algorithm, a set of standard metrics was employed. All experiments were conducted under identical environmental conditions and parameter settings for both the SC-based and CSC-based methods, ensuring a fair comparison focused solely on the effect of descriptor structure.

**Root Mean Square Error (RMSE):** The overall localization accuracy was evaluated using the Root Mean Square Error (RMSE) between the estimated position and the ground-truth trajectory. This metric represents the average localization deviation along the entire path and was computed in the UTM-K coordinate frame. Minimum, mean, and maximum position errors, as well as axis-wise statistics (x,y,z), were also reported. RMSE is defined as

(10) $$\mathrm{RMSE}=\sqrt{\frac{1}{N}\sum_{i=1}^{N}{∥p_{i}^{\mathrm{est}}-p_{i}^{\mathrm{GT}}∥}_{2}^{2}},$$

where $p_{i}^{\mathrm{est}}$ and $p_{i}^{\mathrm{GT}}$ denote the estimated and ground-truth positions, respectively.

**Top-*K* Accuracy:** This metric represents the probability that the correct match is included among the Top-*K* retrieved candidates for each query scan [20]. Top-*K* Accuracy was computed for k={1,5,10}, providing a measure of the retrieval success rate and search reliability in descriptor-based localization.

**Precision–Recall (PR) Curve:** The discriminative capability of the descriptors during global candidate retrieval was evaluated using the PR curve. A retrieved candidate was considered correct if the distance error between the estimated and ground-truth positions was within a predefined threshold ($t_{D}$, in meters). Precision and Recall were plotted over varying distance thresholds, and the Average Precision (AP)—computed as the Area Under the Curve (AUC)—was used as an integrated measure of retrieval performance [1].

**Detection Recall (DR) Curve:** The DR curve was used to assess the relationship between descriptor similarity scores and actual localization success. For each query, the highest similarity score among correct matches was recorded, and Recall was measured over different score thresholds. This analysis quantifies how consistently the descriptor’s similarity score correlates with true spatial correspondence, thereby reflecting the reliability of similarity-based localization.

Overall, these metrics—RMSE, Top-*k* Accuracy, PR, and DR—were employed to comprehensively evaluate the accuracy, retrieval reliability, and robustness of the proposed CSC-based localization framework.

### 4.4. Localization Performance Evaluation

This section evaluates the localization performance of the proposed Cylindrical Scan Context (CSC) in comparison with the conventional Scan Context (SC). The experiments include Precision–Recall (PR) curves, Detection Recall (DR) curves, RMSE-based pose error, and Top-*K* retrieval accuracy, using both the self-collected Pohang datasets (D1_M2, D2_M1) and the MulRan–KAIST datasets (KAIST1–KAISTMAP2, KAIST3–KAISTMAP2).

For the self-collected dataset, the ground-truth correctness threshold was set to 1 m, and approximately 700 query scenes were used for evaluation. For the MulRan–KAIST dataset, a wider correctness threshold of 3 m was applied due to the large-scale campus environment and inherent trajectory variability, and approximately 1200 query scenes were used in the experiments. These settings ensure a fair and environment-appropriate comparison between SC and CSC.

**Figure 6:**
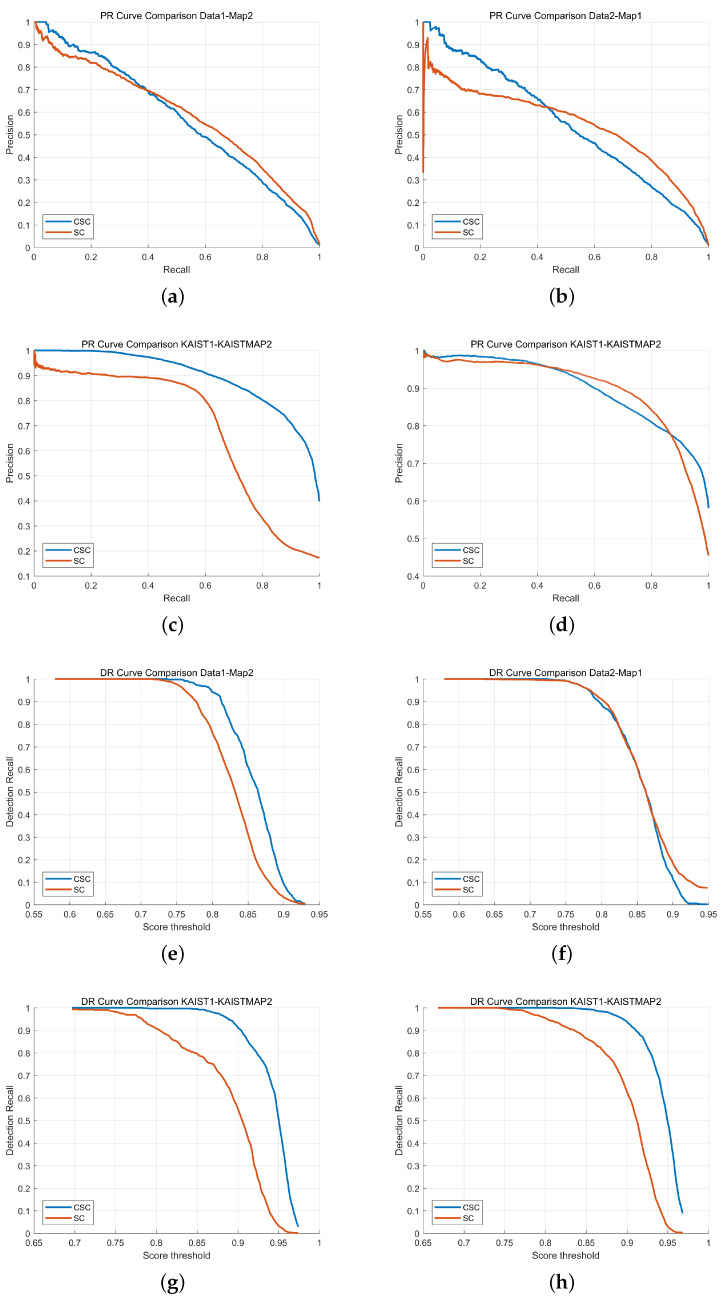
Precision–Recall (PR) curves: (**a**) D1_M2, (**b**) D2_M1, (**c**) KAIST1–KAISTMAP2, (**d**) KAIST3–KAISTMAP2. Detection Recall (DR) curves: (**e**) D1_M2, (**f**) D2_M1, (**g**) KAIST1–KAISTMAP2, (**h**) KAIST3–KAISTMAP2.

The PR curve results shown in Figure 6a–d exhibit distinct characteristics depending on the dataset. For the self-collected D1_M2 pair, SC provides slightly higher precision in the mid-recall range (0.2–0.6), yielding a marginally larger AP. However, CSC becomes comparable as recall approaches 1. In contrast, for the D2_M1 case, CSC maintains consistently higher precision across almost the entire recall range, indicating that CSC is more robust to trajectory differences and environmental variations. For the MulRan–KAIST datasets, CSC demonstrates a significant advantage: in KAIST1–KAISTMAP2, CSC maintains near-perfect precision up to the mid-recall region, while SC shows a steep degradation. In KAIST3–KAISTMAP2, SC performs better in the mid-recall range, but CSC surpasses SC at higher recall levels, demonstrating more stable retrieval as similarity thresholds become strict. Although SC shows higher precision in some mid-recall regions—particularly in short-range or nearly identical revisit conditions—CSC demonstrates more stable performance overall across datasets, especially under viewpoint variation, elevation differences, and heterogeneous traversal paths.

The DR curve results in Figure 6e–h reinforce this observation. In D1_M2, CSC maintains higher recall than SC for score thresholds above 0.85, showing that CSC’s similarity score more reliably correlates with true geometric alignment. In D2_M1, both methods display similar DR behavior, but CSC retains a slight advantage in high-confidence regions. For KAIST1–KAISTMAP2, CSC significantly outperforms SC, maintaining recall above 0.95 over a wide threshold interval, while SC declines sharply. In KAIST3–KAISTMAP2, CSC consistently achieves higher recall across most thresholds, although SC remains competitive in the mid-threshold region. These results confirm that CSC provides more stable and reliable detection performance, especially in large-scale or complex campus environments.

The RMSE-based pose accuracy analysis, summarized in Table 1, further supports the superiority of CSC. For D1_M2, CSC yields a 17% reduction in overall distance RMSE (0.888 m vs. 1.064 m), with substantial reductions in Y- and Z-axis errors by 41% and 42%, respectively. In D2_M1, CSC again achieves slightly lower overall RMSE (1.244 m vs. 1.321 m), with a significant improvement in Y-axis accuracy (0.281 m vs. 0.661 m). CSC also performs strongly on the MulRan–KAIST datasets: in KAIST1–KAISTMAP2, CSC reduces the distance RMSE by approximately 45% (8.4 m vs. 15.113 m), with improvements across all axes. Although KAIST3–KAISTMAP2 shows similar distance RMSE for both methods, CSC maintains more consistent performance on the Z-axis and shows improved lateral accuracy.

Table 2 presents the retrieval accuracy evaluation. For D1_M2, both SC and CSC achieve high Top-*K* accuracy, with SC slightly outperforming CSC at Top-1. However, CSC reaches perfect accuracy (1.000) at Top-10. For D2_M1, SC yields higher Top-1 accuracy, but CSC shows stronger performance at Top-5 and Top-10, indicating that CSC captures more correct candidates when the retrieval window expands. A similar trend is evident in the MulRan–KAIST results: CSC consistently maintains competitive or superior accuracy as *K* increases, demonstrating improved recall and robustness under viewpoint and path variations.

In summary, the experimental results across PR/DR curves, RMSE accuracy, and Top-*K* retrieval demonstrate that CSC provides more stable and reliable localization performance than SC. By preserving vertical structure and offering a more consistent geometric encoding in cylindrical coordinates, CSC enhances both retrieval stability and final pose accuracy in diverse and unstructured outdoor environments. Although SC remains competitive in short-range or nearly identical revisit scenarios, CSC proves to be the more robust choice for real-world applications involving temporal changes, elevation differences, and heterogeneous trajectories.

### 4.5. Performance Analysis

The overall experimental results demonstrate that CSC provides more consistent and reliable localization performance than SC across diverse conditions. In terms of metric-based evaluation, CSC achieves lower RMSE values—particularly along the Y- and Z-axes—indicating improved spatial alignment when elevation changes or vertical structural variations are present. CSC also shows stronger retrieval stability, with rapidly increasing Top-*K* accuracy as the candidate range expands, suggesting that its cylindrical encoding captures more valid matches even under viewpoint differences.

The PR and DR curves further confirm this trend: CSC maintains higher precision and recall in high-confidence regions, especially in datasets involving trajectory or environmental changes. This indicates that the similarity scores produced by CSC more faithfully reflect true geometric correspondence. Altogether, these observations show that preserving vertical structure and representing the environment in cylindrical coordinates enable CSC to deliver robust LiDAR-only localization performance, particularly in unstructured or dynamically varying outdoor environments.

## 5. Conclusions and Future Works

This work presented a LiDAR-only localization framework based on the Cylindrical Scan Context (CSC), which projects point clouds onto an azimuth–height domain and incorporates multiple physical channels to preserve vertical structural information more effectively than the conventional Scan Context (SC). By separating mapping and localization sessions, the proposed framework was evaluated under realistic GPS-denied conditions using pre-built maps.

Across all datasets, CSC demonstrated improved localization stability and geometric accuracy. In the self-collected experiments, CSC achieved up to 17% lower distance RMSE and a 57% reduction in Y-axis error, while in the MulRan–KAIST dataset, it reduced distance RMSE by approximately 45%. CSC also showed stronger recall growth in Top-*K* retrieval and more stable performance in the high-recall regions of the PR/DR curves, highlighting its robustness to viewpoint changes, elevation differences, and traversal variations. Moreover, the cylindrical representation yielded favorable scalability, offering consistent performance across structurally different datasets with lightweight computational cost.

Despite these improvements, the evaluation remains focused on ground-level outdoor environments, and further validation is required for dense urban, multi-level, or industrial settings. Long-term environmental variations were not explicitly considered, and the mapping stage relied on LIO-SAM with fixed parameters, limiting broader sensitivity analyses.

Future work will include channel ablation and accuracy–complexity analysis, development of adaptive and scalable descriptor variants, and evaluation across more diverse and long-term datasets. Statistical significance testing and confidence-interval reporting will also be incorporated to strengthen the reliability of performance comparisons.

In summary, CSC effectively mitigates structural limitations of SC by preserving vertical geometry and enhancing descriptor consistency under environmental and temporal variations. The results confirm CSC as a practical and scalable solution for LiDAR-only localization in GPS-denied or degraded environments, with strong potential for next-generation autonomous navigation systems.

## Figures and Tables

**Table 1:** RMSE comparison of CSC and SC on self-collected and MulRan–KAIST datasets.

Self-Collected Dataset				MulRan–KAIST Dataset			
Dataset	Axis	CSC	SC	Dataset	Axis	CSC	SC
D1_M2	dist	0.888	1.064	KAIST1_KAISTMAP2	dist	8.400	15.113
D1_M2	x	0.711	0.547	KAIST1_KAISTMAP2	x	5.105	10.590
D1_M2	y	0.356	0.607	KAIST1_KAISTMAP2	y	5.331	11.198
D1_M2	z	0.356	0.681	KAIST1_KAISTMAP2	z	5.059	5.357
D2_M1	dist	1.244	1.321	KAIST3_KAISTMAP2	dist	9.950	9.949
D2_M1	x	0.782	0.589	KAIST3_KAISTMAP2	x	4.744	4.543
D2_M1	y	0.281	0.661	KAIST3_KAISTMAP2	y	5.494	5.180
D2_M1	z	0.925	0.982	KAIST3_KAISTMAP2	z	6.817	7.177

**Table 2:** Top-*K* retrieval accuracy comparison of CSC and SC on self-collected and MulRan–KAIST datasets.

Self-Collected Dataset				MulRan-KAIST Dataset			
Dataset	Top-*K*	CSC	SC	Dataset	Top-*K*	CSC	SC
D1_M2	Top1	0.851	0.886	KAIST1_KAISTMAP2	Top1	0.876	0.861
	Top5	0.989	0.981		Top5	0.964	0.865
	Top10	1.000	0.982		Top10	0.987	0.912
D2_M1	Top1	0.752	0.836	KAIST3_KAISTMAP2	Top1	0.847	0.847
	Top5	0.936	0.903		Top5	0.934	0.913
	Top10	0.948	0.907		Top10	0.949	0.950

## Data Availability

The data supporting the findings of this study are not publicly available due to institutional restrictions and privacy/security considerations.

## Abbreviations

The following abbreviations are used in this manuscript:

LiDAR	Light Detection And Ranging
SC	Scan Context
CSC	Cylindrical Scan Context
SLAM	Simultaneous Localization And Mapping
ICP	Iterative Closest Point
IMU	Inertial Measurement Unit
GPS	Global Positioning System
UTM	Universal Transverse Mercator
RMSE	Root Mean Square Error
PR Curve	Precision–Recall Curve
DR Curve	Detection Recall Curve
